# Enhancing the Adhesive Strength of a Plywood Adhesive Developed from Hydrolyzed Specified Risk Materials

**DOI:** 10.3390/polym8080285

**Published:** 2016-08-08

**Authors:** Birendra B. Adhikari, Pooran Appadu, Vadim Kislitsin, Michael Chae, Phillip Choi, David C. Bressler

**Affiliations:** 1 Department of Agricultural, Food and Nutritional Science, Faculty of Agricultural, Life and Environmental Sciences, University of Alberta, Edmonton, AB T6G 2P5, Canada; badhikar@ualberta.ca (B.B.A.); pooran@ualberta.ca (P.A.); mchae@ualberta.ca (M.C.); 2 Department of Chemical and Materials Engineering, Faculty of Engineering, University of Alberta, Edmonton, AB T6G 1H9, Canada; kislitsi@ualberta.ca (V.K.); pchoi@ualberta.ca (P.C.)

**Keywords:** specified risk materials, peptides, PAE resin, plywood adhesive, lap shear strength

## Abstract

The current production of wood composites relies mostly on formaldehyde-based adhesives such as urea formaldehyde (UF) and phenol formaldehyde (PF) resins. As these resins are produced from non-renewable resources, and there are some ongoing issues with possible health hazard due to formaldehyde emission from such products, the purpose of this research was to develop a formaldehyde-free plywood adhesive utilizing waste protein as a renewable feedstock. The feedstock for this work was specified risk material (SRM), which is currently being disposed of either by incineration or by landfilling. In this report, we describe a technology for utilization of SRM for the development of an environmentally friendly plywood adhesive. SRM was thermally hydrolyzed using a Canadian government-approved protocol, and the peptides were recovered from the hydrolyzate. The recovered peptides were chemically crosslinked with polyamidoamine-epichlorohydrin (PAE) resin to develop an adhesive system for bonding of plywood specimens. The effects of crosslinking time, peptides/crosslinking agent ratio, and temperature of hot pressing of plywood specimens on the strength of formulated adhesives were investigated. Formulations containing as much as 78% (*wt*/*wt)* peptides met the ASTM (American Society for Testing and Materials) specifications of minimum dry and soaked shear strength requirement for UF resin type adhesives. Under the optimum conditions tested, the peptides–PAE resin-based formulations resulted in plywood specimens having comparable dry as well as soaked shear strength to that of commercial PF resin.

## 1. Introduction

Production of wood-based panel products such as plywood, oriented strand boards (OSB), medium-density fibreboards (MDF), particleboards, and hardboards requires the use of wood adhesive binders. In 2013, global wood-based panel production reached 367 million m³ [1], requiring 16.2 megatonnes of wood adhesives and binders [2]. Formaldehyde-based resins such as urea formaldehyde (UF), phenol formaldehyde (PF), resorcinol formaldehyde (RF), melamine formaldehyde (MF), and melamine–urea–formaldehyde (MUF) resins are currently the most common type of wood adhesives. Of these, urea–formaldehyde products accounted for over 30% of the global wood adhesives consumed in 2013 [2]. The use of wood adhesive resins is on the rise due to increasing demand of engineered wood products [1]. Accordingly, consumption of formaldehyde-based resins is also on the rise. However, one of the most serious issues with the use of formaldehyde-based resins is the emission of formaldehyde, a known carcinogen [3], from such products. Therefore, there is an increasing concern with regards to possible formaldehyde emission during manufacturing of formaldehyde-based resins and also from the products made using such resins. Consequently, a growing interest lies on the development of formaldehyde-free adhesive systems for production of bonded wood products.

Specified risk materials (SRM) are proteinaceous wastes that represent a significant proportion of slaughterhouse by-products [4]. SRM includes the bovine tissues in which specific misfolded proteins, also known as prions, are likely to concentrate. Such prions are known to cause a neurodegenerative disease called Bovine Spongiform Encephalopathy (BSE) or ”Mad Cow Disease” in cattle [5,6]. Implementation of enhanced feed bans in 2007 in Canada restricted the use of SRM, as well as any tissues that come in contact with SRM, in any food, feed, and fertilizer industry [7]. Consequently, approximately three hundred thousand tonnes of SRM is generated annually in Canada that is either disposed of in landfills or incinerated [8]. In the United States, more than seven hundred thousand tonnes of SRM are being landfilled annually [9] with severe environmental and economical impacts. As proteins are natural polymers containing a variety of functional groups that can interact with wood through a number of physicochemical interactions, they are attractive feedstocks for design and formulation of formaldehyde-free, environmentally-friendly adhesive systems [10,11,12,13,14]. Hence, SRM represents a substantial amount of potentially cheap, abundant, and renewable resource of protein for wood adhesive formulations.

The Canadian Food Inspection Agency (CFIA) recommends landfilling, on-site burial, incineration, composting, and thermal or alkaline hydrolysis as safe ways of SRM disposal [6,15]. Of these methods, thermal or alkaline hydrolysis is an attractive method as it offers a way for processing and recovery of protein from the hydrolyzate, and the recovered protein can be subsequently utilized in a number of value-added applications. Protein recovered from SRM hydrolyzates has shown great promise for development of bioplastics [16] and biocomposites [17]. Very recently, we reported on the utilization of SRM hydrolyzed protein for development of adhesive systems for plywood [18] and oriented strand boards [19]. The formulated adhesives exhibited promising adhesive properties under dry conditions, but the resulting wood composites had limited moisture resistance. 

The potential of SRM protein in wood adhesive formulations can be enhanced if the limited water resistance can be overcome. Similar to other proteins, the SRM hydrolyzates contain a number of reactive functional groups such as amine, carboxyl, sulfahydroxyl, and phenolic hydroxyl, which can be utilized for subsequent chemical modification to enhance adhesive strength as well as water resistance. Water resistance can be enhanced either by chemical modification of end functional groups and/or by chemical crosslinking using a suitable crosslinking reagent. Chemically crosslinked proteins have shown enhanced adhesive strength as well as moisture resistance properties in comparison to their non-crosslinked analogues [10,11,12,14,20]. Glutaraldehyde [14], methylene diphenyl isocyanate (MDI) [10,19], poly(ethylene imine) (PEI) [13], epoxy resin [11], and polyamidoamine-epichlorohydrin (PAE) resin [12,20] are some of the protein crosslinking agents that have shown promise for formulation of formaldehyde-free protein-based wood adhesive systems. 

**Figure 1:**
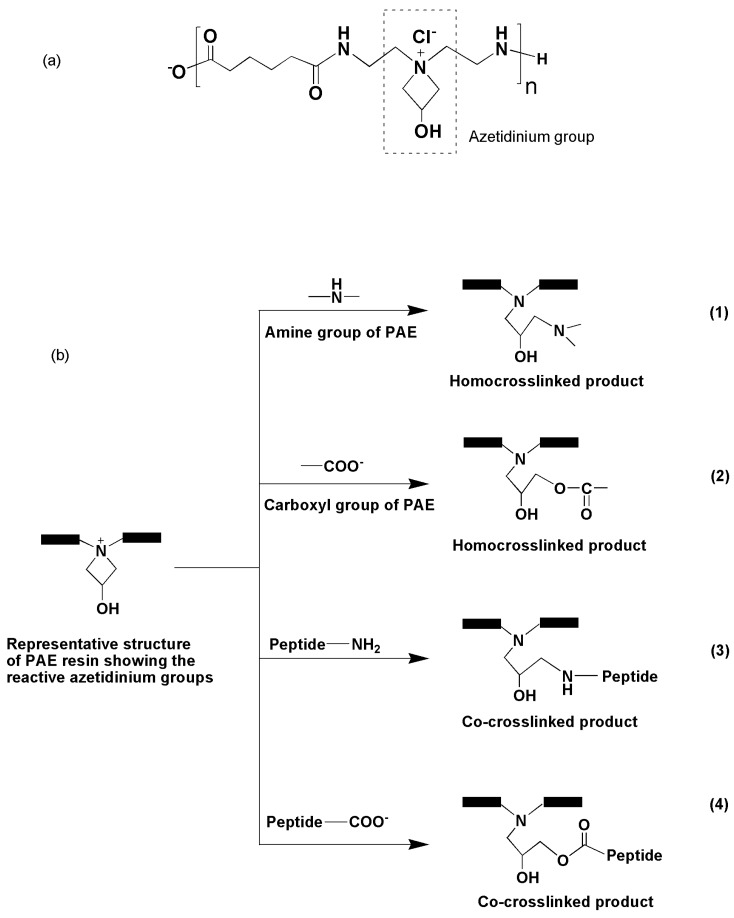
Chemical structure of PAE resin (**a**); and plausible chemical reactions occurring during chemical crosslinking of PAE resin with peptides (**b**). Reactions (1) and (2) represent self-crosslinking reactions of PAE molecules, which occur due to the reactions of the azetidinium groups with secondary amines (Reaction (1)) as well as terminal carboxylate groups (Reaction (2)) of PAE producing a homocrosslinked polymer [12,20,21,22,23]. Co-crosslinking of PAE resin and peptides occurs due to the reactions of azetidinium groups of the resin with amine (Reaction (3)) and carboxylate (Reaction (4)) groups of the peptides [12,20].

PAE resins are water soluble polymeric materials possessing cationic azetidinium groups as reactive sites in the polyamideamine backbone (Figure 1a) [21,22] and are widely used for wet strength enhancement of papers [21,22,23]. These azetidinium groups are susceptible to react with active hydrogen containing groups such as amine, hydroxyl, and carboxyl [12,21,22,23]. In wet strength enhancement of papers, the azetidinium groups of PAE react with carboxylate groups of pulp resulting in a crosslinked product [23]. PAE resin has also shown promise as a crosslinking agent for adhesive development from soy protein isolate [12,20]. In fact, the mixture of soy protein and PAE resin has been used as a commercial binder for production of composite wood products [24]. There are several plausible reactions that can occur during chemical crosslinking of PAE resin molecules and peptides (Figure 1b), which can all contribute to enhanced adhesive strength and water resistance of peptides-based adhesive systems. Here, we report the potential application of PAE resin for crosslinking of SRM hydrolyzates for formulation of formaldehyde free plywood adhesive, and the effects of various parameters in adhesive strength and water resistance property of the formulated adhesive.

## 2. Materials and Methods

### 2.1. Materials and Chemicals

SRM samples were handled according to CFIA protocols for safe handling and disinfection of SRM material [8]. The PAE resin used in this study was Kymene™ 557H resin, which was kindly supplied by Solenis (Wilmington, DE, USA). The phenol formaldehyde (PF) resin was sourced from a commercial supplier. As per the information provided by the suppliers, the total solid content of PAE resin was 12.5%, and that of PF resin was 43%. The poplar veneers used in this study were purchased from a local supplier. The veneer sheets were rotary peeled. Filter paper (Whatman no. 4, pore size 20–25 mm) was sourced from Whatman (Cambridge, UK). Hexane (99.9%, ACS grade) was acquired from Fisher Scientific (Fair Lawn, NJ, USA). 

### 2.2. Methods

#### 2.2.1. SRM Hydrolysis and Recovery of Peptides

A thermal hydrolysis procedure that was previously developed in our lab was used in this study, which was based on a Canadian Food Inspection Agency (CFIA) approved protocol for SRM disposal [8,15]. Hydrolysis of SRM was performed in an enclosed pressure vessel at a temperature of ≥180 °C and pressure of ≥1200 kPa for 40 min per cycle. When necessary, the required pressure was achieved by adding nitrogen gas in to the reactor vessel. In a typical hydrolysis run, a mixture of 1 kg each of SRM and distilled water was enclosed in a 5.5 L stainless steel reactor vessel (Parr 4582, Parr Instrument Company, Moline, IL, USA) and then subjected to hydrolysis. During hydrolysis, the SRM and water were mixed at an agitation speed of 200 rpm. The reaction was controlled by Parr reactor controller (Parr 4848, Parr Instrument Company, Moline, IL, USA). The potentially hazardous SRM sample (before hydrolysis) was handled in a government approved biosafety level II containment lab. Surfaces were decontaminated with 5% Environ LpH for 30 min followed by 70% ethanol.

The post hydrolysis material is considered non-hazardous, and was processed by following general laboratory practices. The procedure developed by Mekonnen et al. was adopted for recovery of proteinaceous fragments from the hydrolyzate [8]. In a typical run, 450 mL of distilled water was added to 100 g of SRM hydrolyzate, the mixture was agitated at 200 rpm in a shaker (New Brunswick™ Innova® 44, New Brunswick Scientific Company Inc., Edison, NJ, USA) for 10 min, and then allowed to settle for 10 min. The aqueous portion was then subjected to centrifugation (Avanti J-26 XP high-performance centrifuge, Beckman Coulter Canada LP, Mississauga, ON, Canada) at 7000× *g* for 40 min to remove insoluble residues. The supernatant was then subjected to vacuum filtration (Whatman 4 filter paper) to remove any remaining insolubles. The collected filtrate was then washed with hexane to remove any lipids. The aqueous fraction was then lyophilized to get a proteinaceous cake (35 ± 1% yield from the feed material). The recovered protein hydrolyzates are referred to as peptides hereafter. 

#### 2.2.2. Characterization of Peptides

##### Estimation of Carboxyl Groups

The estimated number of carboxyl groups in the recovered peptides was estimated by following an adopted pH titration method reported elsewhere [25,26]. Typically, 0.33 g peptides were solubilized in 50 mL Milli-Q water, with the resulting solution having a pH of 5.60. Under constant stirring, small aliquots of 0.1 M NaOH were added to the peptide solution to bring the pH to 7.0. At this pH, all the carboxyl groups are converted to carboxylate anion, and the peptides are converted to a zwitterionic form -OOC-R-NH_3_^+^. Small aliquots of 0.1 M HCl were then added to the solution to bring the pH to 3.0 at which the carboxylate anions are protonated, and the peptides are converted to the protonated form HOOC-R-NH_3_^+^. Quantification of carboxyl groups was then performed under the assumption that the amount of HCl necessary to change the pH from 6.0 to 3.0 is directly related to the number of carboxyl groups. The titration experiments were conducted in triplicates. 

##### Estimation of Amine Groups

Amine groups present in the recovered peptides were estimated using an *o*-phthaldehyde (OPA) method, which measures UV absorbance of the resulting complexes [26,27]. An OPA solution was prepared as follows: 50 mL of 0.1 M sodium borate solution, 5.0 mL of 20% (*w*/*w*) sodium dodecyl sulphate (SDS), 2 mL of OPA solution (40 mg OPA/mL methanol) and 0.2 mL of β-mercaptoethanol were mixed in a 100 mL volumetric flask, and the final volume was adjusted to 100 mL by adding Milli-Q water. The peptide solution was prepared by dissolving peptides (1 g/L) in 12.5 mM sodium borate buffer. UV-vis absorbance measurement of the peptide solution was done as follows: 50 µL of peptide solution was added to 1.0 mL of OPA reagent. The samples were vigorously mixed and incubated for 10 min at room temperature. Then, the absorbance of the samples was read at 340 nm using Milli-Q water as a blank. A calibration curve was obtained in a similar way, but using l-leucine. All the experiments were conducted in triplicate. 

### 2.3. Adhesive Formulation

#### 2.3.1. General Method 

The peptides–PAE adhesive was prepared by mixing the peptides with PAE resin, and stirring the resulting slurry at room temperature. The adhesive formulation containing of 20% solid could be spread reasonably well, and had ability to properly wet the wood surface. Hence, the total solid content of our adhesive formulations was 20%. In a typical experiment, the adhesive was prepared by mixing 20.0 g PAE resin (total solids = 2.5 g) with 1.88 g peptides (formulation 6, Table 1), which corresponds to a peptides:PAE resin ratio of 1:1.33 on dry weight basis. 

#### 2.3.2. Varying the Effect of Crosslinking Ratio

The optimum ratio of peptides and crosslinking agent was explored by varying the amounts of PAE resin and peptides in the mixture as shown in Table 1. The total solid content of these formulations was 20%, and the weight percentage of crosslinking agent was varied from zero (peptides only) to 57%. 

### 2.4. Preparation of Plywood Specimens

Plywood specimens were prepared as follows: poplar veneers of 1.6 mm thickness were cut into panels of 50 mm by 20 mm dimensions, and pre-conditioned at a relative humidity of 70% to reach a moisture content of 10%–12%, which was determined using an oven drying method. A constant amount of adhesive slurry (3.0 mg·cm^−2^ adhesive on dry weight basis) was then applied on to 20 mm × 20 mm area of one end of the wood pieces in such a way that it properly wet the surface. The adhesive coated wood pieces were assembled in such a way that the grains of two pieces were oriented in parallel. The assembled pieces were allowed to equilibrate for 15 min to allow the adhesive to penetrate into the wood, and then subjected to hot pressing (Carver Inc., Wabash, IN, USA) at a temperature of 120 °C and pressure of 3.5 MPa for 5 min. The hot pressing temperature was varied in experiments investigating the effect of pressing temperature. The resulted plywood specimens had a lap area of 20 mm × 20 mm.

### 2.5. Testing of Plywood Specimens

After hot pressing, the plywood specimens were conditioned for 7 days in a temperature–humidity chamber maintained at 50% relative humidity and 23 °C. The lap shear strength of the plywood specimens was then tested according to ASTM standard method D2339-98 [28] using a mechanical test system (MTS 810, MTS Systems Corporation, Eden Prairie, MN, USA) equipped with a 10 kN load cell. The loading rate was at a crosshead speed of 1 mm·min^−1^. The force required to shear the glued wood pieces was measured by pulling them apart from the two edges, and was expressed as adhesive strength (MPa) after dividing the force (N) by the glued area (mm^2^). The data for each sample were obtained from an average of six specimens. 

The water resistance of adhesive systems were evaluated by testing soaked shear strength of plywood specimens according to ASTM standard method D1183-03 (test designation C) [29]. The plywood specimens were soaked in tap water at 23 °C for 48 h, which was followed by conditioning at 23 °C and 50% humidity for 7 days in a humidity chamber before evaluating the lap shear strength. 

## 3. Results and Discussion

### 3.1. Characterization of Peptides

SRM, the primary feedstock for our adhesive formulations, was subjected to thermal hydrolysis, followed by extraction and purification of the resulting peptides, using procedures developed in our laboratory [8]. In order to acquire a better understanding of the functional groups available in the recovered peptide material, we assessed the number of free carboxylic acid and amine groups. These groups are the primary functionalities of interest for our adhesive formulations, with regards to chemical modification and/or crosslinking. The number of mmoles of –COOH groups, estimated by pH titration [25,26], was found to be 1.66 mmoles/g peptides. Furthermore, the number of mmoles of –NH_2_ groups, determined using the OPA method [26,27], was found to be 0.60 mmoles/g peptides. This indicates that the amine groups are present in lower numbers than the carboxyl groups in the bulk material of hydrolyzed peptides. These results are likely attributable to the phenomenon of deamination of protein hydrolyzate as was observed in the case of thermal hydrolysis of bovine serum albumin (BSA) [30,31,32] and hydrothermal treatment of dewatered sewage sludge [33]. During thermal hydrolysis of proteins, the polypeptide intermediate products are further hydrolyzed to amino acids. The amino acids are labile under subcritical hydrothermal conditions, and undergo hydrothermolysis producing short chain fatty acids [30,31,32]. Together, these phenomena result in a decrease in the number of amine groups and an increase in the number of carboxyl groups in the bulk material.

### 3.2. Effect of Various Factors on Lap Shear Strength of Plywood Specimens

Thermal hydrolysis denatures proteins, producing small protein fragments and exposing specific functional groups. When incorporated directly into adhesive formulations, these relatively low molecular weight polymers display poor adhesive strength [10]. Additionally, the SRM hydrolyzates have inherently high hydrophilicity [8], which translates to low water resistance of the adhesive [18,19]. To alleviate these concerns, we focused our efforts on chemically crosslinking the hydrolyzed peptides in order to generate a better adhesive system. We examined the effect of different factors—reaction time, ratio of peptides and PAE resin, and curing (hot pressing) conditions—that influence the extent of crosslinking between peptides and PAE resin, as well as the mechanical strength of the resulting adhesive formulation.

#### 3.2.1. Effect of Crosslinking Time

**Figure 2:**
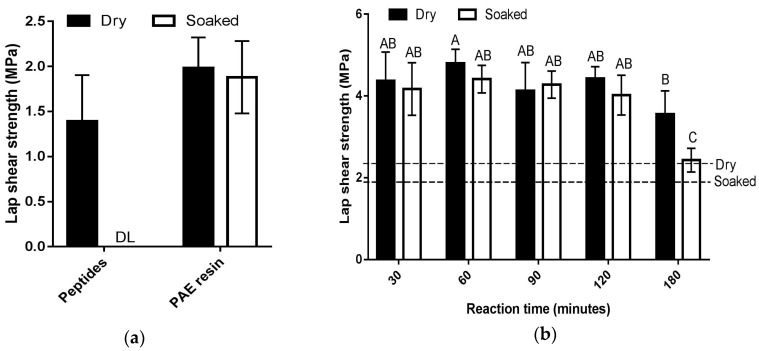
Lap shear strength of plywood specimens bonded with peptides or PAE resin alone (**a**); or peptides–PAE adhesives crosslinked for various amounts of time (**b**). For peptides–PAE formulations, 1.88 g of peptides were mixed with 20.0 g PAE resin (peptides: PAE resin = 1:1.33 on dry weight basis). Specimens were hot pressed at 120 °C and 3.5 Mpa for five min. Error bars indicate standard deviation of six plywood specimen measurements. Some specimens delaminated when soaked in water (DL). Means that do not share a letter are significantly different (Tukey, 95% confidence level). The minimum shear strength requirements as specified by ASTM D4690 are shown: 2.344 MPa for dry shear strength; and 1.93 MPa for soaked shear strength [34].

First, we examined the dry and soaked shear strengths of plywood specimens bonded with peptides or PAE resin alone as a control. Using peptides alone, an average dry shear strength of 1.39 ± 0.51 MPa was observed, and all of the specimens were delaminated upon soaking in water (Figure 2a). On the other hand, the plywood specimens bonded with the PAE resin alone yielded an average dry shear strength of 1.98 ± 0.33 MPa, and soaked shear strength of 1.88 ± 0.40 MPa (Figure 2a). Next, we prepared the peptides–PAE adhesive system and investigated the effect of crosslinking time of peptides and PAE resin on dry and soaked shear strengths of plywood specimens bonded with the resulting formulation. As expected, the peptides–PAE combination exhibited notable improvement in lap shear strength in dry as well as soaked conditions as compared to the individual components (Figure 2b). This is attributable to the combined effect of chemical crosslinking of peptides and PAE resin molecules, and chemical interactions of the crosslinked product with the functional groups of the cellulose. Chemical crosslinking leads to the formation of rigid, three-dimensional networks of polymers connected through covalent linkages, and prevents the polymer chains from creeping during mechanical testing. Additionally, chemical crosslinking imparts water resistant properties to the polymers, and by extension, to the adhesive formulation. Furthermore, similar to what PAE does in wet strength enhancement of papers [22], the crosslinked product of PAE resin and peptides would react with the functional groups of cellulose (wood) in the curing step, resulting in formation of water resistant, covalent bonds [12,20]. Together, these phenomena resulted in enhanced lap shear strength in dry as well as soaked conditions. 

The results of the lap shear strengths of the peptides–PAE adhesive as a function of crosslinking time indicate that effective crosslinking of peptides and PAE resin was achieved within 30 min of mixing, and there were no significant differences in dry and soaked shear strength among the 30-, 60-, 90-, and 120-min treatments (Figure 2b). Furthermore, all four treatment times resulted in adhesive formulations that met the minimum shear strength requirement specifications of ASTM D4690 for urea formaldehyde resin type wood adhesives: 2.344 MPa for dry shear strength, and 1.93 MPa for soaked shear strength [34]. Considering these specifications as a baseline for performance evaluation of new adhesive formulations, the peptides–PAE formulations safely passed the ASTM requirement for UF resin type of wood adhesives implying that this new adhesive system has tremendous potential to substitute UF-based resins for wood bonding applications. A drop in performance was observed for the formulation crosslinked for 180 min (Figure 2b). The reason for this is currently unknown and will be the focus of future research.

One of the requirements of commercial adhesives is that they should have a reasonable pot life, which is the length of time during which it remains workable without substantial loss of adhesive strength. For plywood applications, for example, adhesives should cure slowly at room temperature, allowing sufficient time for adhesive coating and assembling of wood components, and the adhesive should set quickly upon heating (hot pressing) of the wood specimens [35]. The results shown in Figure 2b indicate that the peptides–PAE adhesive system fulfills this requirement under the specified experimental conditions. This adhesive can be used up to 120 min after mixing of the adhesive components without significant reduction in adhesive strength and water resistance property. Consequently, a stirring time of 120 min was considered the pot life of this adhesive, and all other experiments were conducted using this crosslinking time.

#### 3.2.2. Effect of Crosslinking Ratio

**Figure 3:**
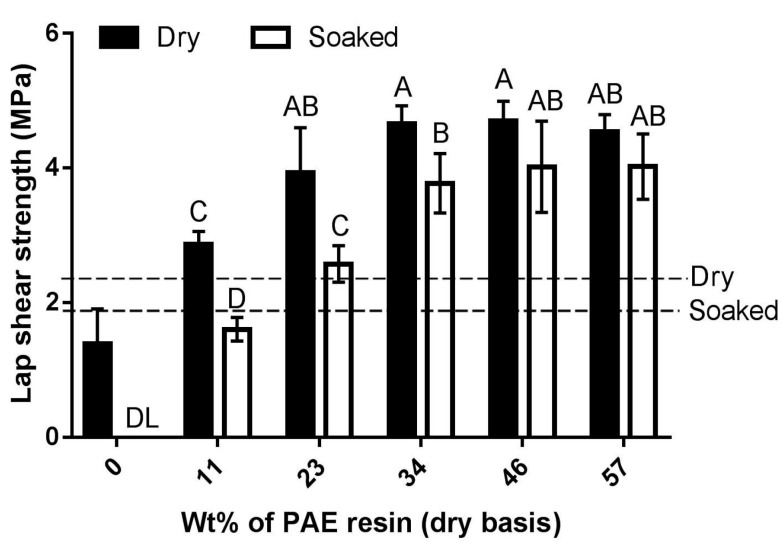
Effect of the weight ratio of peptides and PAE on lap shear strength of plywood specimens bonded with the peptides–PAE adhesive. Specimens were crosslinked for 120 min, and then hot pressed at 120 °C and 3.5 MPa for five min. Error bars are standard deviation of six plywood specimen measurements. Specimens that delaminated when soaked in water are indicated (DL). Means that do not share a letter are significantly different (Tukey, 95% confidence level). The minimum shear strength requirements as specified by ASTM D4690 are shown: 2.344 MPa for dry shear strength; 1.93 MPa for soaked shear strength [34].

In two-component adhesive systems, curing agents or hardeners are the chemicals that react with the resin in stoichiometric proportions to produce three-dimensional rigid networks of thermosetting resins after curing. In our formulation, the peptides and PAE resin react in specific proportions, producing a thermosetting adhesive resin after curing. Figure 3 shows the effect of weight ratio (on dry basis) of PAE resin and peptides on lap shear strength of bonded plywood specimens. 

The results indicate that the strength properties of the peptides–PAE system were significantly affected by the crosslinking ratio. At low crosslinking levels (i.e., lower levels of PAE), the resulting formulations had moderate lap shear strength and limited water resistance. However, the lap shear strength and water resistance of the peptides–PAE adhesive system improved progressively with increasing proportions of crosslinking agent before tapering off when PAE levels reached 34%. This implies that the amount of crosslinking agent in formulations with PAE levels of 23% or lower was limiting, and all of the peptides were not effectively crosslinked. This also implies that any of the unreacted peptides of formulations consisting of 11% and 23% PAE resin were presumably washed off during the soaking test, resulting in significantly lower soaked shear strength as seen in Figure 3. Conversely, further addition of crosslinking agent after 34% did not cause notable changes in strength property of the peptides–PAE formulations most likely because the crosslinked density of polymers did not increase significantly after 34 wt % of crosslinking agent. 

The formulation containing 34% PAE resin and 66% peptides contained 1.5 g PAE resin and 2.9 g peptides (Table 1). Based on the molecular weight of a repeating unit of PAE resin (332.82 amu; see Figure 1a for the structure of PAE), this formulation contains 4.5 mmoles of azetidinium groups. On the other hand, from our estimation of carboxyl and amine groups, this formulation contains 4.8 mmoles of carboxyl groups and 1.9 mmoles of amine groups. The pH of the peptides–PAE formulation was 5.0 at which the amine groups exist in their protonated form, and are not expected to contribute greatly in crosslinking reactions. As the number of mmoles of azetidinum groups and carboxyl groups are roughly in 1:1 proportion, it also seems reasonable from the perspective of number of mmoles of reacting functional groups that the crosslinked density did not increase significantly after 34 wt % of crosslinking agent. 

Considering the ASTM specification requirements, formulations containing 23–57% PAE resin safely passed the requirements of UF type of wood adhesive resins for both dry as well as soaked conditions. Analysis of results of Figure 3 further indicates that even though the soaked shear strengths of formulations containing 34, 46 and 57% PAE resin were not significantly different from each other, the dry and soaked shear strengths of formulation containing 34% PAE resin were, however, significantly different from each other. Hence, the formulation consisting of 46% PAE resin and 54% peptides, for which the soaked shear strength is statistically similar to the dry shear strength, was considered for further investigations. 

#### 3.2.3. Effect of Hot Pressing Temperature

**Figure 4:**
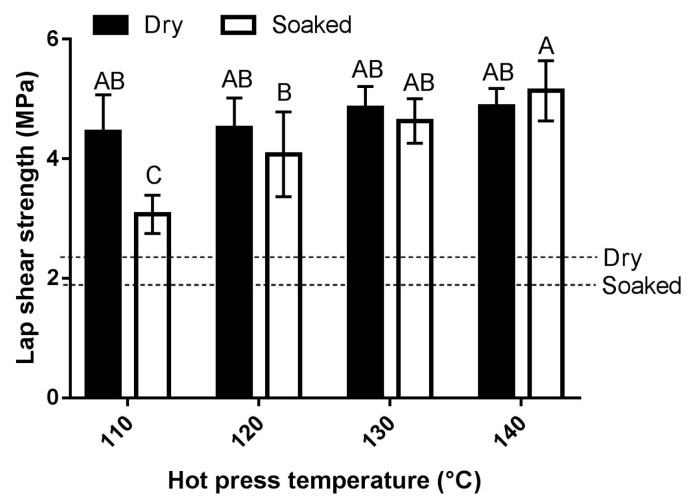
Effect of hot press temperature on lap shear strength of plywood specimens bonded with the peptides–PAE adhesive. For these experiments, a formulation consisting of 46% PAE resin and 54% peptides was used after crosslinking for 120 min. Specimens were hot pressed at 3.5 MPa for five min. Error bars are standard deviation of six plywood specimen measurements. Means that do not share a letter are significantly different (Tukey, 95% confidence level). The minimum shear strength requirements as specified by ASTM D4690 are indicated.

In adhesive bonding of wood, temperature has a marked effect on the crosslinking density of the resulting thermosetting polymer, water evaporation from the bondline, and interaction between the adhesive and the substrate [13,20,36,37,38]. The widely used hot press temperature in commercial production of wood composites is in the range of 120 to 160 °C [13]. For plywood production, typical press temperatures range from 107 to 135 °C for hardwood plywood and from 132 to 165 °C for softwood plywood [39]. As poplar is considered hardwood, the effect of hot press temperature for making plywood using our peptides–PAE adhesive and poplar veneer was studied in the temperature range of 110 to 140 °C. The results showing the effect of hot pressing temperature on lap shear strength of bonded plywood specimens are presented in Figure 4.

Hot pressing temperatures in the range of 110 to 140 °C had no effect on dry shear strength of plywood specimens as the results were not significantly different (Figure 4). Conversely, pressing temperature had a remarkable effect on the soaked shear strength of plywood specimens with higher temperatures generally yielding specimens with higher lap shear strength (Figure 4). The significant effect of pressing temperature on soaked shear strength was also supported by the mode of failure of test specimens observed during mechanical testing (Table 2).

In gluing of wood specimens by hot pressing, increasing the hot pressing temperature would have two marked effects: (i) the viscosity of the adhesive system would be lowered, which leads to enhanced penetration ability of the adhesive and increased effective surface area of interaction of the adhesive and substrate; and (ii) the possibility of chemical interactions in the adhesive formulation as well as in the interface would be increased. Both of these phenomena would lead to enhanced soaked shear strength of the adhesive system as observed in the results of Figure 4. Even though a similar effect of hot press temperature would be expected on dry shear strength as well, this was not the case (Figure 4). The failure mode of test samples (Table 2) describes the case of dry shear strength. The test samples under dry conditions achieved bond strength equal to that of wood strength at 120 °C, and there was no significant effect of hot pressing temperature upon further increasing the temperature since the strength of wood limits the strength of the adhesive system. It also appears that the test specimens hot pressed at 110 °C had achieved bond strength very close to that of wood strength under dry conditions. Since the hot pressing pressure of 3.5 MPa is likely to exceed the compression strength (perpendicular to grain) of poplar veneer sheets, it is probable that the fibres were slightly deformed during sample preparation and the percentage wood failure presented in Table 2 was somehow compromised due to the effect of pressing pressure. An increase in soaked shear strength of bonded wood specimens with increasing hot pressing temperature was also observed for a soy protein adhesive employed for making fibreboards [38], a guanidine hydrochloride-modified soy protein-based wood adhesive [37], and a soy protein–melamine–urea–formaldehyde adhesive system for wood binding applications [40]. Curing temperature was also found to be the most influential factor in soaked shear strength of PUF resin bonded wood specimens [36]. 

### 3.3. Comparison of Adhesive Strength with Commercial PF Resin

**Figure 5:**
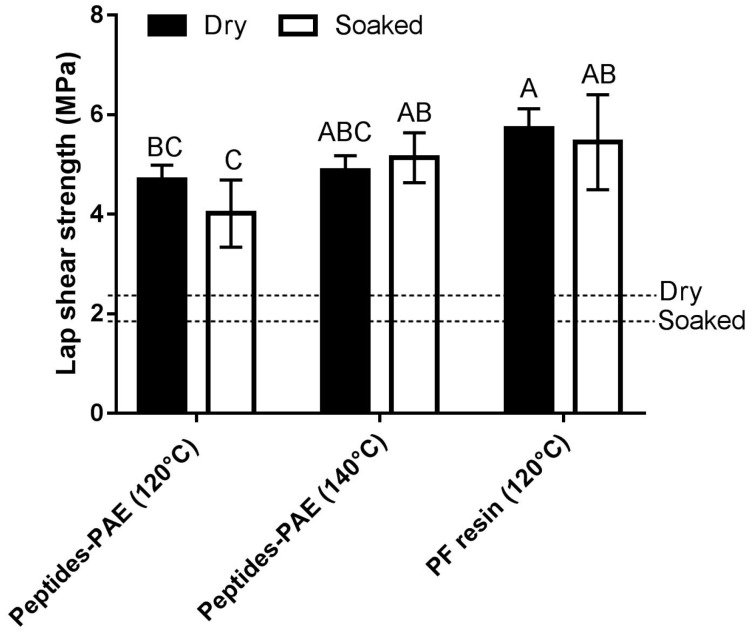
Comparison of adhesive performance of peptides–PAE resin to that of a commercially-available phenol–formaldehyde (PF) resin. Specimens were hot pressed at 3.5 MPa for five min at hot pressing temperature shown in parenthesis. Error bars are standard deviation of six plywood specimen measurements. Means that do not share a letter are significantly different (Tukey, 95% confidence level). The minimum shear strength requirements as specified by ASTM D4690 are indicated.

The strength properties of our peptides–PAE resin adhesive system were compared to that of commercial PF resin. Upon hot pressing at 120 °C and 3.5 MPa for five min, the dry and soaked shear strengths of the peptides–PAE adhesive were significantly lower than that of the PF resin (Figure 5). Furthermore, all the samples prepared by using PF resins underwent cohesive wood failure, which was not the case for the peptides–PAE formulation at 120 °C hot pressing. However, as already stated, the test specimens underwent 100% cohesive wood failure when the samples using the peptides–PAE resin adhesive were hot pressed at 140 °C. The lap shear strengths of plywood specimens and failure mode indicated that the peptides–PAE system demonstrated comparable performance to that of PF resin at elevated pressing temperature. While the dry shear strength of plywood specimens prepared by using peptides–PAE formulation at 140 °C was comparable to that of PF resin, the soaked shear strength was statistically similar to that of PF resin. As the plywood samples hot pressed at 120 °C using PF resin underwent 100% cohesive wood failure, an increase in adhesive strength would not be observed on increasing the hot pressing temperature and thus the PF resin was not studied at a hot pressing temperature of 140 °C.

## 4. Conclusions 

Here, we show the successful incorporation of peptides derived from thermally hydrolyzed SRM in adhesive formulations using PAE resin as a crosslinking agent. The effect of adhesive formulation parameters such as crosslinking time, ratio of crosslinking agent and peptides, and hot pressing temperature were examined. The peptides and PAE resin can be crosslinked for 30 to 120 min at room temperature with no significant difference in dry or soaked shear strength, which means this formulation has a suitable pot life for plywood applications. Peptides–PAE adhesive formulations containing 23 to 57% PAE met the ASTM D4690 requirements of both dry and soaked shear strengths for urea formaldehyde resin type of wood adhesive. Hot pressing temperature was shown to have significant impact on soaked shear strength. At 140 °C, plywood specimens fabricated using the peptides–PAE adhesives displayed dry as well as soaked shear strength comparable to that of a commercial PF resin. Overall, we have successfully overcome the issues of limited water resistance of SRM peptides as plywood adhesive that we had encountered in our previous studies, and have demonstrated a platform for commercial utilization of SRM for value-added applications.

## Figures and Tables

**Table 1:** Formulation of peptides–PAE adhesive by varying the ratio of peptides and PAE resin.

	PAE resin (g)	Peptides (g)	Water (g)	Total weight (g)	Solid from PAE resin, dry basis (g) ^1^	Total solid (g)	Solid content (%)	Wt % of PAE resin (dry basis)
1	0.0	4.4	17.5	21.9	0.00	4.4	20.0	0.0
2	4.0	3.9	14.0	21.9	0.50	4.4	20.0	11.4
3	8.0	3.4	10.5	21.9	1.00	4.4	20.0	22.8
4	12.0	2.9	7.0	21.9	1.50	4.4	20.0	34.3
5	16.0	2.4	3.5	21.9	2.00	4.4	20.0	45.7
6	20.0	1.9	0.0	21.9	2.50	4.4	20.0	57.1

^1^ Total solid content of PAE resin is 12.5%.

**Table 2:** Mode of failure of dry and soaked samples hot pressed at different temperatures. For each condition, seven test specimens were examined.

Press temp (°C)	Dry samples	Soaked samples
110	adhesive failure ^1^ in 86% of samples, fibre pulled out ^2^ in 14% of samples	100% samples underwent adhesive failure
120	cohesive wood failure ^3^ in 43% of samples, fibre pulled out in 57% of samples	adhesive failure in 57% of samples, fibre pulled out in 43% of samples
130	cohesive wood failure in 86% of samples, fibre pulled out in 14% of samples	cohesive wood failure in 86% of samples, fibre pulled out in 14% of samples
140	cohesive wood failure in 86% of samples, fibre pulled out in 14% of samples	100% samples underwent cohesive wood failure

^1^ Failure within the bulk of adhesive; ^2^ shallow wood failure with wood fibres remaining attached to the adhesive film; and ^3^ deep wood fracture from the bondline.
